# Adult-onset deactivation of autophagy leads to loss of synapse homeostasis and cognitive impairment, with implications for alzheimer disease

**DOI:** 10.1080/15548627.2024.2368335

**Published:** 2024-07-01

**Authors:** Hilary Grosso Jasutkar, Elizabeth M. Wasserlein, Azeez Ishola, Nicole Litt, Agnieszka Staniszewski, Ottavio Arancio, Ai Yamamoto

**Affiliations:** a Department of Neurology, Columbia University, New York, NY, USA; b Department of Neurology, Rutgers Robert Wood Johnson Medical School, Piscataway, NJ, USA; c Doctoral Program in Neurobiology and Behavior, Columbia University, New York, NY, USA; d The Taub Institute for Research on Alzheimer’s Disease and the Aging Brain, Columbia University, New York, NY, USA; e Department of Pathology and Cell Biology, Columbia University, New York, NY, USA

**Keywords:** Alzheimer disease, autophagy, cognition, hippocampus, synapse homeostasis

## Abstract

A growing number of studies link dysfunction of macroautophagy/autophagy to the pathogenesis of diseases such as Alzheimer disease (AD). Given the global importance of autophagy for homeostasis, how its dysfunction can lead to specific neurological changes is puzzling. To examine this further, we compared the global deactivation of autophagy in the adult mouse using the *atg7*iKO with the impact of AD-associated pathogenic changes in autophagic processing of synaptic proteins. Isolated forebrain synaptosomes, rather than total homogenates, from *atg7*iKO mice demonstrated accumulation of synaptic proteins, suggesting that the synapse might be a vulnerable site for protein homeostasis disruption. Moreover, the deactivation of autophagy resulted in impaired cognitive performance over time, whereas gross locomotor skills remained intact. Despite deactivation of autophagy for 6.5 weeks, changes in cognition were in the absence of cell death or synapse loss. In the symptomatic APP PSEN1 double-transgenic mouse model of AD, we found that the impairment in autophagosome maturation coupled with diminished presence of discrete synaptic proteins in autophagosomes isolated from these mice, leading to the accumulation of one of these proteins in the detergent insoluble protein fraction. This protein, SLC17A7/Vglut, also accumulated in *atg7*iKO mouse synaptosomes. Taken together, we conclude that synaptic autophagy plays a role in maintaining protein homeostasis, and that while decreasing autophagy interrupts normal cognitive function, the preservation of locomotion suggests that not all circuits are affected similarly. Our data suggest that the disruption of autophagic activity in AD may have relevance for the cognitive impairment in this adult-onset neurodegenerative disease. **Abbreviations**: 2dRAWM: 2-day radial arm water maze; AD: Alzheimer disease; Aβ: amyloid-beta; AIF1/Iba1: allograft inflammatory factor 1; APP: amyloid beta precursor protein; ATG7: autophagy related 7; AV: autophagic vacuole; CCV: cargo capture value; Ctrl: control; DLG4/PSD-95: discs large MAGUK scaffold protein 4; GFAP: glial fibrillary acidic protein; GRIN2B/NMDAR2b: glutamate ionotropic receptor NMDA type subunit 2B; LTD: long-term depression; MAP1LC3/LC3: microtubule associated protein 1 light chain 3; m/o: months-old; PNS: post-nuclear supernatant; PSEN1/PS1: presenilin 1; SHB: sucrose homogenization buffer; SLC32A1/Vgat: solute carrier family 32 member 1; SLC17A7/Vglut1: solute carrier family 17 member 7; SNAP25: synaptosome associated protein 25; SQSTM1/p62: sequestosome 1; SYN1: synapsin I; SYP: synaptophysin ; SYT1: synaptotagmin 1; Tam: tamoxifen; VAMP2: vesicle associated membrane protein 2; VCL: vinculin; wks: weeks.

## Introduction

The homeostatic pathway macroautophagy, herein referred to as autophagy, has been implicated in a large number of diseases, including neurodegenerative conditions such as Alzheimer disease (AD) [1]. Given that cells of the central nervous system such as neurons are highly compartmentalized, understanding how autophagy functions within discrete compartments may provide an important foundation for studying these devastating conditions. A neuronal compartment in which autophagy plays a specialized role is the synapse. The presence of a high density of proteins and the relatively long distance between the synapse and the soma puts the synapse at particularly high risk for the accumulation of old or damaged proteins and organelles [2]. Autophagic vacuoles (AVs), the organelles in which autophagic cargos are trafficked to the lysosome, can form locally at the axon terminal [3–8], and multiple synaptic proteins have been implicated as targets of autophagy [8–21].

Synaptic loss is an early finding in AD pathogenesis [22–29], and thus the role of autophagy at the synapse may be especially relevant to this most prevalent neurodegenerative disease. In the United States alone, there were approximately 6.2 million Americans age 65 and older living with this disease in 2021 [30], and this number balloons to approximately 32 million persons when considering the worldwide prevalence [31]. Studies describe autophagy dysfunction in both AD patient samples [32,33] and in mouse models of the disease [34–38], with the most notable change described as the accumulation of AVs, strongly suggesting the dysfunction is in the ability of autophagosomes to mature. Nonetheless, more upstream changes have been noted, including altered expression levels of BECN1/Beclin 1 [39] and other autophagy related genes [38]. In addition, direct association of autophagy dysfunction and pathogenic proteins associated with AD, namely amyloid-beta (Aβ) and hyperphosphorylated MAPT/tau, suggest that dysfunction in AD might have unique mechanisms relative to other degenerative diseases. For example, Aβ interferes with retrograde transport of autophagosomes in the axons [33], leading to an accumulation of AVs [40–42], whereas hyperphosphorylated MAPT/tau colocalizes with the autophagosome marker MAP1LC3/LC3 in postmortem patient brain tissue [43], and interferes with autophagosome-lysosome fusion [44]. Nonetheless, given the many pathologic changes present in an AD brain, whether this disruption in autophagic function contributes to disease pathogenesis or is a later consequence of degenerative changes is still unclear. Moreover, although studies clearly indicate autophagy is essential for synaptic function and brain health [45–50], given that the outright disruption of autophagy can lead to widespread changes in the brain [45–47,49], including outright degeneration of the Purkinje cells of the cerebellum [45,47,49], and autophagy dysfunction has been implicated across very different neurodegenerative diseases [51], whether the loss of autophagy can drive specific, disease-relevant changes for any given disease, such as cognitive dysfunction in the aging population, is very much uncertain.

Here, we test whether a disruption of autophagy in the adult brain might capture those pathogenic changes associated with AD. To do so, we examined an inducible knockout model of *Atg7* (*atg7*iKO) to ask whether the deactivation of autophagy throughout the adult brain leads to changes in synaptic homeostasis and cognition, and then to determine whether similar synaptic changes and autophagy deficits are observed in a mouse model of AD. We find that the deactivation of autophagy in the adult brain can lead to synaptosome-specific accumulation of synaptic proteins that are masked when studying whole tissue homogenates. This accumulation, but not cell or synapse loss, was sufficient to be associated with impaired cognitive performance in two different tasks, the Barnes Maze and Morris Water Maze. Notably, cognition was selectively impaired since locomotor behavior remained intact, suggesting a selective vulnerability of discrete circuits to autophagy deficits. Next, in the brains of the APP PSEN1 double transgenic mouse model of AD, the abnormal accumulation of AVs is accompanied by decreases in the relative presence of discrete synaptic proteins in isolated AD autophagosomes, leading to an accumulation of synaptic protein in the detergent insoluble fraction. Taken together, these data reveal that global autophagy dysfunction in adult mice can perturb synaptic homeostasis, and preferentially cognitive function, and may therefore contribute to the early changes associated with disorders of cognition such as AD.

## Results

### Changes in autophagic activity can be detected in synaptosomes

**Figure 1.**
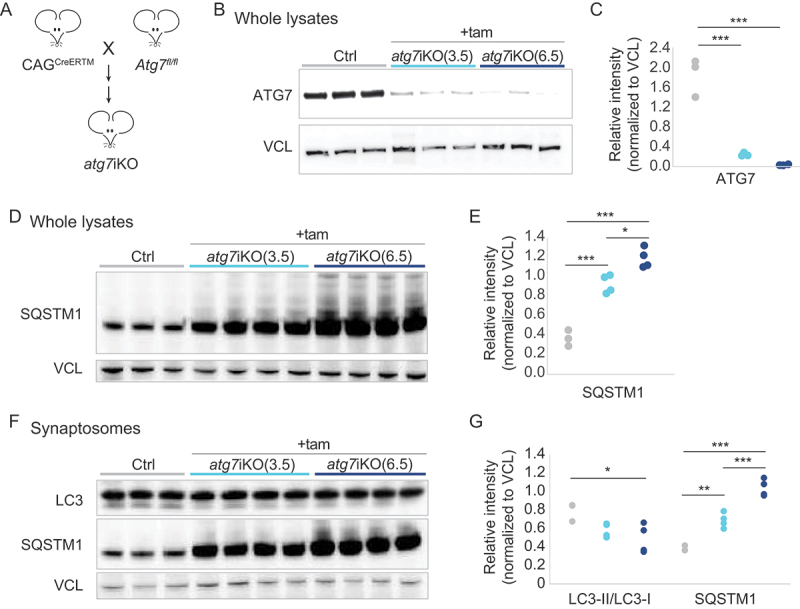
Inactivation of ATG7 in adult mice. (A) Schematic representation of creation of atg7iKO mice. (B-E) Forebrains of Control (Ctrl) or atg7iKO mice 3.5 or 6.5 wks after tam injection (+tam) were homogenized and subjected to quantitative western blot and probed for (B and C) ATG7 and (D and E) SQSTM1/p62. (F and G) Quantitative immunoblot analyses against MAP1LC3/LC3 or SQSTM1/p62 of synaptosomal fractions from mouse forebrain. VCL was used as a loading control. Ctrl, n = 3; atg7iKO(3.5), n = 4; atg7iKO(6.5), n = 4. Statistical analyses are found in Table S1. *p < 0.05, **p < 0.01, ***p < 0.001.

Studies indicate that autophagy can initiate locally at the synapse and may play a vital role in maintaining synaptic function [51,52]. Given the importance of autophagy in neurodevelopment [51], we evaluated the impact of autophagy deactivation on synaptic protein homeostasis and cognitive function by generating an inducible model with which we can deactivate autophagy in adult mice. These *atg7*iKO mice were created by crossing mice carrying a conditional *Atg7* allele [53] with mice carrying a tamoxifen (tam)-inducible Cre under the chicken *ACTB* (actin beta) promotor (CAG^CreERTM^) [54] (Figure 1A), similar to a line created by crossing conditional *Atg7* mice with UBC^CreERT2^ [55]. Autophagy was deactivated in adult mice by inducing *Cre*-mediated excision in 4- to 5-months-old (m/o) mice, which were then sacrificed 3.5 or 6.5 weeks (wks) later. Immunoblot analyses reveal that already after 3.5 wks, ATG7 protein levels were significantly reduced (Figure 1B,C), and a proxy for autophagic activity, SQSTM1/p62 (sequestome 1) levels, also showed accumulation, confirming autophagy deactivation in *atg7*iKO mice (Figure 1D,E,S1A,B).

Previous studies have shown that autophagosomes can be identified at the synapse [5,6,8,21,33,56–58], suggesting that this process can initiate locally. To confirm this finding biochemically, we isolated synaptosomes using two independent protocols (Figure 1F,G, S1C-G), and probed for SQSTM1/p62 and MAP1LC3/LC3 (microtubule associated protein 1 light chain 3). As suggested by prior studies, SQSTM1/p62 and LC3-II were present in synaptosomes from control (Ctrl) mice (Figure 1F,G, S1G). Similarly to whole lysates, upon depletion of ATG7, SQSTM1/p62 began to accumulate significantly in synaptosomes by 3.5 wks post-KO, and continued to increase into 6.5 wks (Figure 1F,G). Notably, a reduced level of LC3-II persisted in synaptosomes, significantly declining only at 6.5 wks. Given the loss of ATG7 expression at 3.5 wks, and the clear rise in SQSTM1/p62 accumulation, these data suggest that the persistence of LC3-II may reflect a delay in the maturation of AVs or recycling of LC3-II from membrane, both of which are ATG7-independent events.

### Distinct differences in synaptic protein accumulation are present in the synapse upon deactivation of autophagy

**Figure 2.**
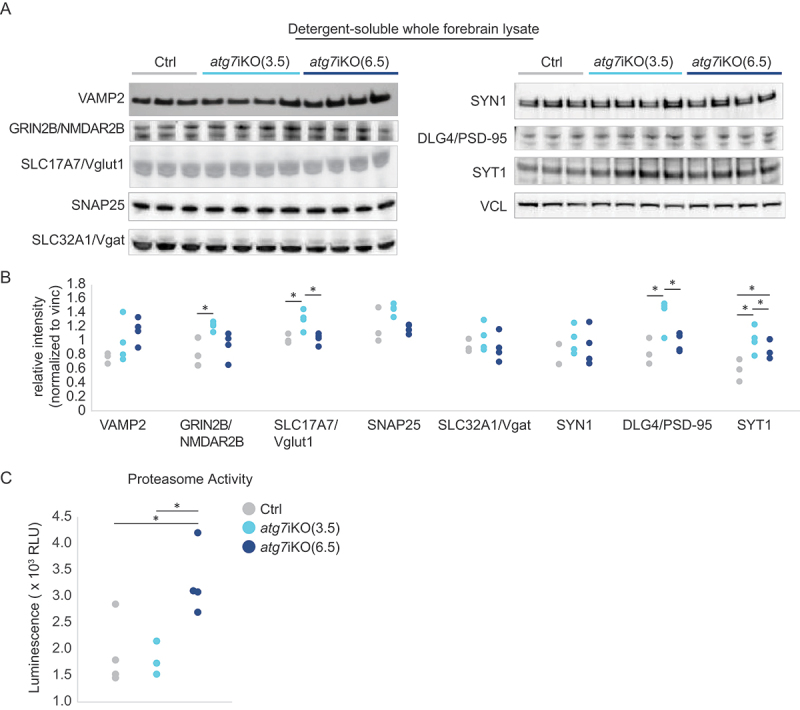
Analyses of whole tissue homogenates suggests that cells compensate for autophagy deactivation over time. (A and B) Quantitative western blot analyses of detergent soluble lysates of mouse forebrain. Analyses reveal a significant accumulation of synaptic proteins GRIN2B/NMDAR2B, SLC17A7/Vglut1, DLG4/PSD-95, and SYT1 in atg7iKO(3.5) mice, which in atg7iKO(6.5) mice, return to Ctrl levels. VAMP2, SLC32A1/Vgat and SYN1, did not show any change. VCL was used as a loading control. Ctrl, n = 3; atg7iKO(3.5), n = 4; atg7iKO(6.5), n = 4. (C) Chymotrypsin-like activity of the proteasome in forebrain tissue of mice. atg7iKO(6.5) mice demonstrate a significant increase in proteasome activity. Ctrl, n = 3; atg7iKO(3.5), n = 4; atg7iKO(6.5), n = 4. Statistical analyses are found in Table S1. *p < 0.05.

To better understand the importance of autophagy in degrading synaptic proteins, we next performed quantitative western blot analysis of forebrain homogenates of *atg7*iKO mice (Figure 2, S2). We found that at 3.5 wks there was a significant accumulation of GRIN2B/NMDAR2b (glutamate ionotropic receptor NMDA type subunit 2B), SLC17A7/Vglut1 (solute carrier family 17 member 7), DLG4/PSD-95 (discs large MAGUK scaffold protein 4), and SYT1 (synaptotagmin 1), as well as a trend toward an increase (overall significant difference, but post-hoc analysis did not find significant differences between groups, Table S1) of SNAP25 (synaptosome associated protein 25) in the detergent-soluble fraction of the whole forebrain homogenate (Figure 2A,B). This effect was specific for these proteins, as other proteins tested, such as SLC32A1/Vgat (solute carrier family 32 member 1) and SYN1 (synapsin I), did not show a difference. These data suggest that the disruption of autophagy can immediately impact the steady state levels of some, but not all, synaptic proteins. Notably, the vesicle transporters, SLC17A7/Vglut1 and SLC32A1/Vgat, were affected differently by the loss of autophagy, suggesting that a neuronal subtype-specific reliance may be present. Although protein accumulation can often disrupt protein folding, the increased accumulation at 3.5 wks was only observed in the detergent-soluble fraction and not the insoluble one (Figure S2), suggesting that protein folding remains intact. Surprisingly, similar analyses of forebrain homogenates 6.5 wks post-KO revealed that protein accumulation was resolved, potentially reflecting how different protein trafficking pathways may be able to compensate for the loss of autophagy (Figure 2A,B). To investigate this further, we performed a proteasome activity assay that monitored chymotrypsin-like activity (Figure 2C). We found that activity remained at control levels 3.5 wks after autophagy deactivation, but was significantly increased by 6.5 wks after autophagy deactivation. This increased proteasome activity suggests that other degradative pathways can respond to autophagy deactivation in the brain, which may account for the normalization of protein levels in the detergent-soluble fraction. RT-PCR confirmed that the observed differences were not due to changes in expression of these transcripts (Table S2).

**Figure 3.**
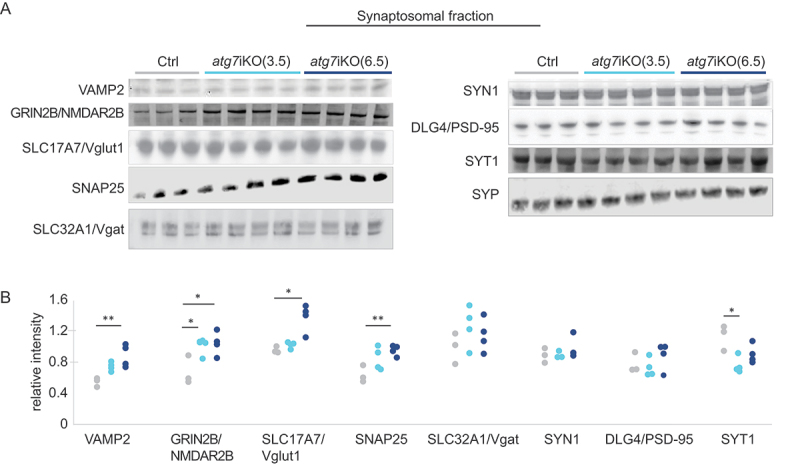
Unlike whole brain homogenates, synaptic protein accumulation following autophagy deactivation is observed in synaptosome preparations (A and B) Quantitative western blot analyses of the synaptosomal fraction from atg7iKO mouse forebrain. SYP (synaptophysin) is used as a loading control. Ctrl, n = 3; atg7iKO(3.5), n = 4; atg7iKO(6.5), n = 4. Statistical analyses are found in Table S1.*p < 0.05, **p < 0.01.

Given the compartmentalized nature of neurons, we next determined if our observations in the forebrain homogenates accurately reflected the state of the synaptic compartment by probing for the same proteins in isolated synaptosomes from these mice. These studies revealed considerable differences between the forebrain homogenates (Figure 2) and the synaptosomes (Figure 3). In contrast to forebrain homogenates, at 3.5 wks there was only a significant increase in GRIN2B/NMDAR2b relative to controls, but not in any of the other proteins assessed. However, by 6.5 wks, the levels of VAMP2 (vesicle associated membrane protein 2), GRIN2B/NMDAR2b, SLC17A7/Vglut1, and SNAP25 had all increased relative to control levels. SYT1 showed decreased levels relative to controls at 3.5 wks and no difference at 6.5 wks in the synaptosomal fraction. Consistent with forebrain homogenates, SLC32A1/Vgat and SYN1 levels remained unchanged at both time points in the synaptosomal fraction, again suggesting that the function of autophagy to process synaptic proteins may have neuronal subtype specificity. Taken together, these data revealed notable compartment specific changes in protein accumulation upon the deactivation of autophagy. Moreover, the greater, cumulative accretion of proteins in the synaptic compartment at 6.5 wks, despite the increase in proteasomal activity at that age (Figure 2C), suggests that protein homeostasis at the synapse may be more reliant upon autophagy than other subcellular compartments.

### Synapse numbers and cell numbers in the hippocampus are not affected by the absence of autophagy

**Figure 4.**
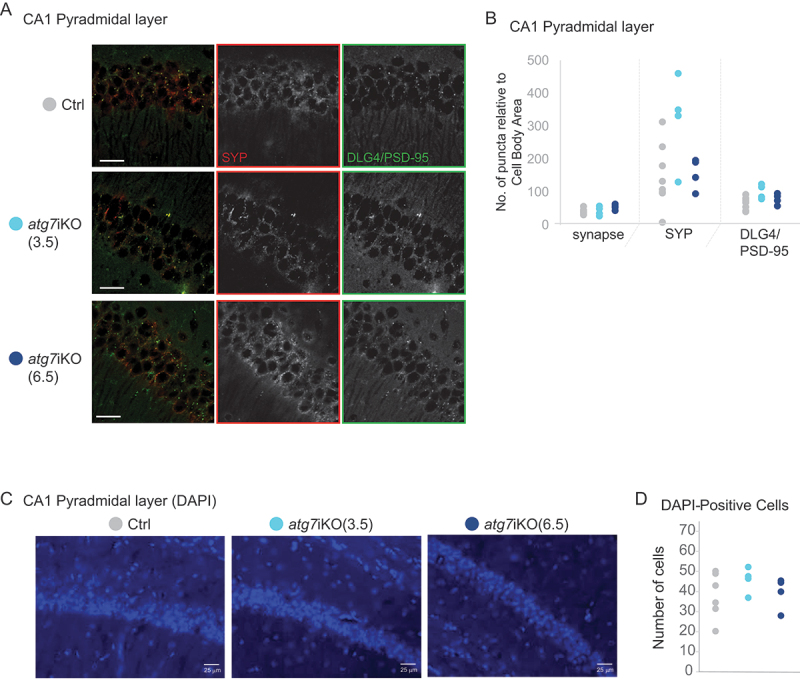
Deactivation of autophagy in adult mice does not affect synapse or cell numbers. (A and B) Immunofluorescence of coronal sections of the CA1 region of the hippocampus, stained for SYP (green) and DLG4/PSD-95 (red) in the pyramidal layer of CA1. (B) Colocalization between SYP^+^ and DLG4/PSD-95^+^ puncta is used to define a synapse. Ctrl, n = 8; atg7iKO(3.5), n = 4; atg7iKO(6.5), n = 4. (C and D) DAPI staining of the same hippocampal region. (D) Number of cells per unit area. Scale bar: 25 µm. Statistical analyses are found in Table S1.

We next determined if the disruption of protein homeostasis at the synapse led to synapse loss. We therefore performed immunofluorescence imaging of the CA1 region of the hippocampi of *atg7*iKO mice 3.5 wks or 6.5 wks post-deactivation. To identify synapses, co-immunostaining of SYP as a presynaptic marker and DLG4/PSD-95 as a post-synaptic marker was used. We did not see any change in synapse numbers between any of the groups in the pyramidal layer of CA1 of the hippocampus, as measured by colocalization of SYP and DLG4/PSD-95 signal (Figure 4A,B).

Autophagy is an essential pathway [53], and previous studies have shown that different neuronal subtypes are preferentially vulnerable or resilient to the loss of autophagy [51]. Thus, we also assessed the hippocampus for cell death by measuring the number of DAPI-positive cells in the pyramidal layer of CA1 in *atg7*iKO mice 3.5 or 6.5 wks post-KO. As suggested by the similarity in synapse numbers, we found no cell loss at either time point following autophagy deactivation, indicating that within the 6.5 wk timeframe that we evaluated, the loss of autophagy does not lead to cell death in CA1 (Figure 4C,D).

### Deactivation of autophagy does not lead to an inflammatory response in the hippocampus

**Figure 5.**
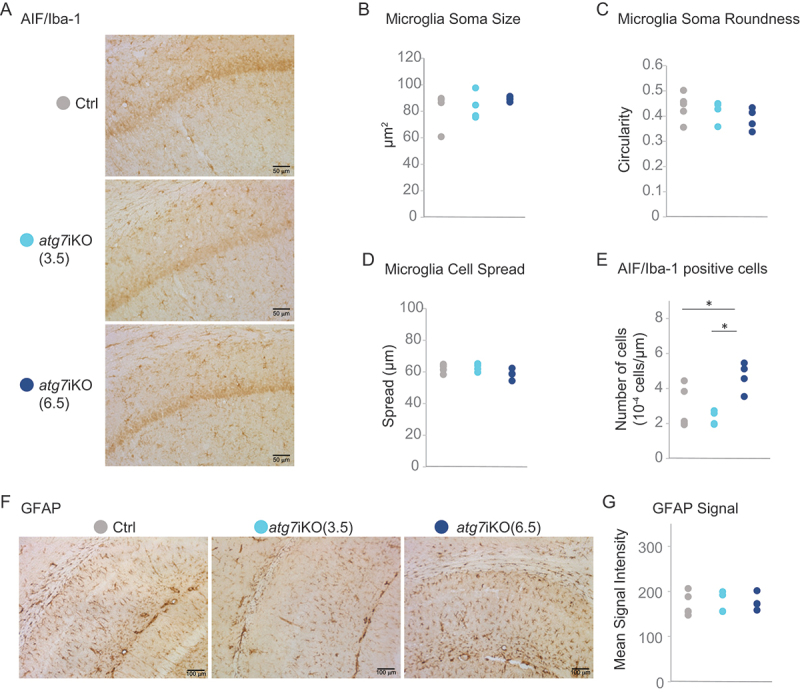
Deactivation of autophagy does not result in neuroinflammation in the hippocampus. (A-E) Immunohistochemistry of coronal brain sections of hippocampus stained for AIF1/Iba1 as a marker for microglia. AIF1^+^ cells were assessed for (B) soma size, (C) soma roundness, (D) cell spread and (E) the total cell number. Scale bar: 50 µm (F and G) Staining against GFAP as a marker for astrocytes (G) Quantification for GFAP signal intensity. Scale bar: 100 µm. Ctrl, n = 5–6; atg7iKO (3.5 wks), n = 4; atg7iKO (6.5 wks), n = 4. Statistical analyses are found in Table S1. *p < 0.05.

Autophagy is also an important metabolic pathway within glial cells [59–62], and our autophagy deactivation paradigm was not cell-type specific. Therefore, to investigate if our findings could be mediated by an effect of autophagy deactivation on microglia or astrocytes, we performed immunostaining for AIF1/Iba1 (allograft inflammatory factor 1) and GFAP (glial fibrillary acidic protein), respectively, in CA1 of *atg7*iKO mice. We assessed microglial morphology with respect to cell roundness, cell body size, and cell spread – all markers of microglial reactivity [63]. We found no differences in microglial morphology by any of these parameters at any time point, suggesting that autophagy deactivation does not induce a change in microglial reactivity (Figure 5A–D). However, although the microglia did not have a morphology suggestive of increased activation, we did see an increased number of microglia in CA1 of the hippocampus at 6.5 wks, as measured by cell number per area (Figure 5A,E). This may reflect an early step in microglia reactivity, although this is controversial [64,65]. We found that GFAP staining intensity was equal in all groups, indicating that there was no difference in astrocyte number or reactivity (Figure 5F,G).

### Deactivation of autophagy leads to deficits in memory-dependent tasks

**Figure 6.**
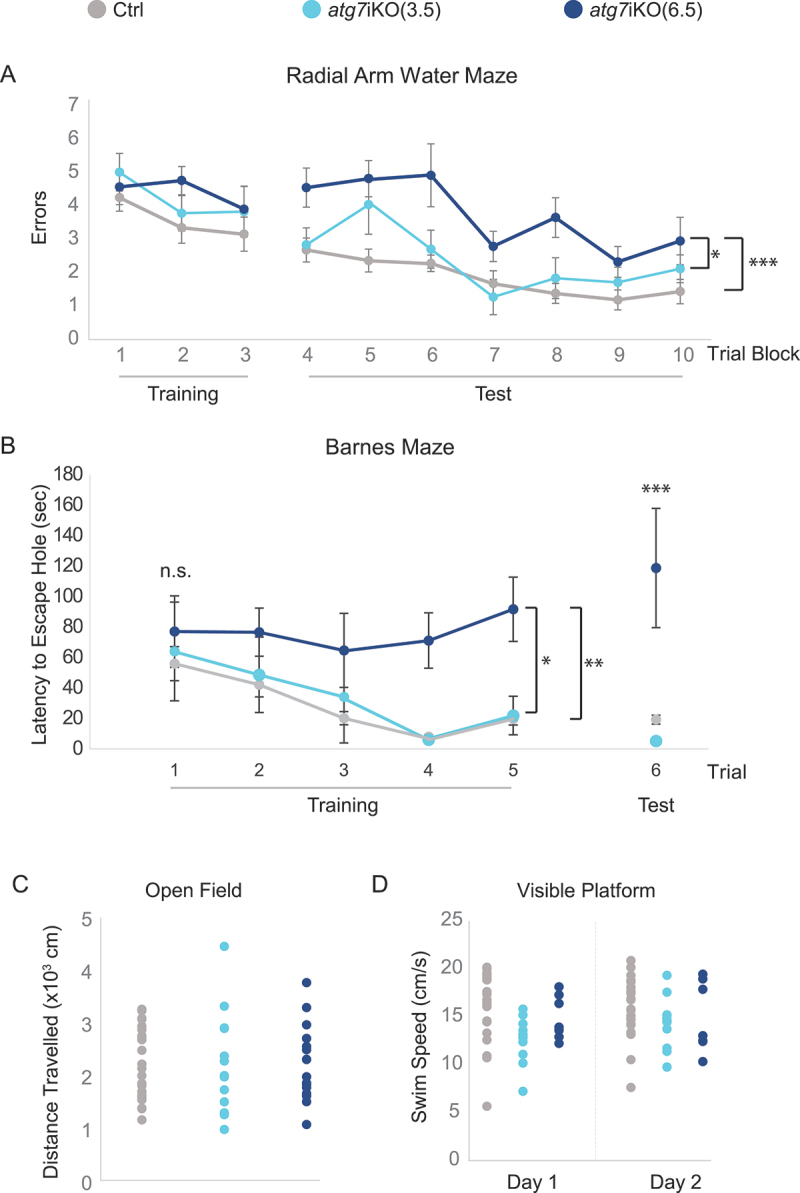
Deactivation of autophagy in adult mice leads to impairment in memory-dependent tasks. (A) 2dRAWM. Over Trial block 1–3 (Training), mice are trained to find a visible platform located in one arm of a 6-arm pool. Mice then must find a hidden platform over Trial blocks 4–5 on day 1, and over Trial blocks 6–10 on day 2. Day 2 is considered the testing day (Test). Although all groups performed similarly during training, only atg7iKO(6.5) mice consistently made more errors than their littermate Ctrls and atg7iKO(3.5) mice during testing. Ctrl, n = 24; atg7iKO(3.5), n = 13; atg7iKO(6.5), n = 10. Mean±St. Dev shown. (B) Barnes maze. Mice are trained to find an escape hole on an open platform over 3 days (Trials 1–5). After a 48-h intertrial interval, they are tested to find the escape hole (Trial 6). On Trials 1–3, all groups exhibited a similar latency to reach the escape hole. With continued Training and on the Test trial, Ctrl and atg7iKO(3.5) were able to acquire and execute the task, whereas atg7iKO(6.5) mice could not acquire the task. Ctrl, n = 16; atg7iKO(3.5), n = 4; atg7iKO(6.5), n = 4. Mean±St. Dev shown. (C and D) Cognitive differences could not be explained by a difference in motor performance. (C) Open Field Maze. Total distance traveled over a 60 min trial. Ctrl, n = 24; atg7iKO(3.5), n = 13; atg7iKO(6.5), n = 10. Individual data points shown. (D) Visible platform test. Daily swim speed average over 6 trials on Day1 and Day 2. Ctrl, n = 23; atg7iKO(3.5), n = 12; atg7iKO(6.5), n = 6. A significant difference in swim speed was found on Day 1 that resolved by Day 2 in the atg7iKO(3.5) group. Statistical analyses are found in Table S1.*p < 0.05, **p < 0.01, ***p < 0.001.

Given that there was no gross pathology observed in the *atg7*iKO hippocampus, we were next able to determine if the changes in protein turnover at the synapse were associated with changes in memory function. The *atg7*iKO mice were tested on the 2-day radial arm water maze test (2dRAWM) [66] and the Barnes maze [67,68] at 2–4 or 5–7 weeks after autophagy deactivation (3.5 wks or 6.5 wks, respectively). On the 2dRAWM, during the testing trials, despite a deactivation of autophagy for 3.5 wks, *atg7*iKO mice were able to find a hidden platform similar to the Ctrls. After 6.5 wks, however, *atg7*iKO mice performed significantly worse (Figure 6A). A similar outcome was observed in the Barnes maze (Figure 6B): After 3.5 wks, mice acquired the ability to find the escape hole similarly to control mice, but after 6.5 wks this was no longer the case. These data suggest that as synaptic proteins accumulate in the synapse, impairments in memory appear. These deficits in the 2dRAWM and Barnes maze were in the absence of locomotor changes: All 3 cohorts of mice demonstrated similar exploratory activity on the open field task, as measured by distance traveled (Figure 6C). On the visible platform task, *atg7*iKO(3.5) mice did demonstrate a decreased swim speed relative to control mice; however, this difference was not seen in *atg7*iKO(6.5) mice (Figure 6D). This normal performance on the visible platform task in the *atg7*iKO(6.5) group suggests that the difference in memory was not due to motor, sensorial, or motivational deficits.

### Altered autophagy in APP PSEN1 double transgenic mice

**Figure 7.**
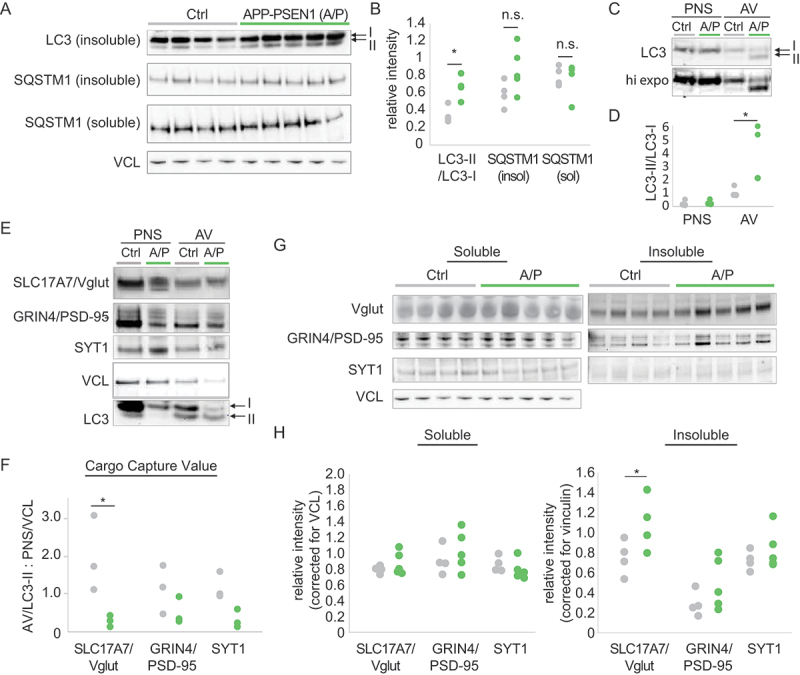
Abnormal autophagic flux in APP PSEN1 mice leads to synaptic protein accumulation. (A and B) Quantitative western blot of Ctrl or APP PSEN1 (A/P) whole forebrain homogenates, fractionated to detergent soluble and insoluble fractions. Blots were probed for MAP1LC3/LC3 or SQSTM1/p62. VCL was used as a loading control. Ctrl, n = 4; A/P, n = 5. Individual data points shown (C-F) Quantitative western blot of AVs isolated from Ctrl or A/P mice and probed for MAP1LC3/LC3. (C) Representative immunoblot for MAP1LC3/LC3. Higher exposure (hi expo) shown to indicate LC3-II levels in the Ctrl AV. (D) LC3-II:LC3-I ratio. (E and F) Quantitative western blot to determine the relative quantity of synaptic proteins cargo such as SLC17A7/Vglut1, DLG4/PSD-95 and SYT1. (F) Cargo capture value (CCV). Represents the relative amount of protein in the AVs, with respect to total protein levels. Protein levels in AVs are normalized to LC3-II, then corrected for the relative amount of the protein in the PNS, normalized to VCL, to account for potential differences in overall protein levels between the two groups. Individual data points for each AV preparation (N) shown. N = 3 AV fractionations/genotype. Each fractionation represents n = 4–5 brains. (G and H) Quantitative western blot of homogenates described in (A), probed for SLC17A7/Vglut1, DLG4/PSD-95, and SYT1. VCL was used as a loading control. Ctrl, n = 4; A/P, n = 5. Individual data points shown. *p < 0.05.

Previous reports have suggested that autophagy is abnormal in patients with AD and there is evidence that gene mutations thought to be causative in AD may contribute to autophagy dysfunction [33,69–71]. However, the process of autophagy is intimately interconnected with other processes implicated in AD such as endocytosis and lysosomal degradation [72]. As a result, it is not clear if autophagy is disrupted as a proximal insult in AD, or indirectly due to disruption in related pathways. Given our findings in the *atg7*iKO mice, we evaluated whether the AD-associated mutations of the APP PSEN1 double transgenic mouse model of AD [73], a model that over-expresses mutant forms of the AD-associated proteins APP (amyloid beta precursor protein) and PSEN1 (presenilin 1), lead to measurable changes in autophagy. We collected forebrain homogenates from 4-month-old (m/o) APP PSEN1 and Ctrl littermate mice, and probed western blots of these homogenates for MAP1LC3/LC3 (Figure 7A,B). We found a significantly greater LC3-II:LC3-I ratio in the detergent-insoluble fraction of forebrain homogenates of the AD mice relative to control animals, suggesting an accumulation of autophagic membranes. Given that there was no increase in LC3-II in the soluble fraction (Figure S4A), this suggests an accumulated form, possibly reflecting the accumulation of AVs observed across different APP PSEN1 models [33,40,42]. Consistent with this was that SQSTM1/p62 levels showed no significant difference suggesting that the packaging of cargo was still occurring and AV formation is intact (Figure 7A,B).

Although the SQSTM1/p62 levels suggested that there was no gross loss of autophagic function, it is possible that the accumulation of AVs may disrupt the turnover of discrete cargoes that might not rely on SQSTM1/p62 for their packaging. To examine this further, we next used our previously described approach [74–77] to enrich for AVs from the brains of the APP PSEN1 mice (Figure 7C,D). Consistent with our findings in homogenates, the LC3-II:LC3-I ratio was significantly higher in APP PSEN1 mice relative to controls already at 4 m/o. Given the proposed role of autophagy in the trafficking of synaptic proteins [78] and our findings in the *atg7*iKO mice, we next probed the isolated AVs for synaptic proteins (Figure 7E,F), focusing on previously reported cargoes DLG4/PSD-95 and SYT1 [8,11,12], and SLC17A7/Vglut1, which we found was sensitive to autophagy modulation (Figures 2, 3) Calculation of a cargo capture value (CCV, Figure 7F) revealed a significantly decreased presence of SLC17A7/Vglut1 from the APP PSEN1brains relative to Ctrl, but not for DLG4/PSD-95 and SYT1. This suggests that in addition to impacting AV maturation, there could also be protein-specific differences in cargo capture in this AD mouse model.

We next asked whether differences in the CCV value were associated with their accumulation. We hypothesized that decreased synaptic proteins in AVs would ultimately lead to an inability to capture and turnover these proteins, and thus, to their abnormal accumulation. To determine this biochemically, we revisited the detergent-soluble and -insoluble forebrain lysates (Figure 7G,H). Consistent with our finding that SYT1 and DLG4/PSD-95 were present in brain AVs from APP PSEN1 mice at levels similar to control and not dependent on autophagy for turnover in the *atg7*iKO mice, there was not an accumulation of either protein in brain homogenates from these animals. In contrast, SLC17A7/Vglut1, which was less present in APP PSEN1 AVs and was dependent upon autophagy for turnover in the *atg7*iKO mice (Figure 3), accumulated in the insoluble fraction from APP PSEN1 brains relative to levels in controls. We also enriched for the synapse and probed western blots of this synaptosomal fraction for DLG4/PSD-95 and SYT1, but found that the quantity of these proteins were not changed in APP PSEN1 mice in that fraction either (Figure S1). Overall, the early accumulation of AVs in the APP PSEN1 mice results in changes in the ability of autophagy to degrade certain but not all synaptic proteins. SLC17A7/Vglut1 being especially sensitive to this loss suggests that unlike other cargoes, which may also utilize additional pathways such as the proteasome or endolysosomal system for degradation [79], some proteins may be especially sensitive to autophagy disruption, which is consistent with our observations in the *atg7*iKO mice. Moreover, given that protein accumulation was sufficient to evoke cognitive changes in the *atg7*iKO mice, these data suggest that the early changes in autophagy observed in the APP PSEN1 mice may be indicative of how a cumulative impact on autophagy may ultimately contribute to the cognitive decline seen in AD.

## Discussion

Autophagy is an essential pathway to maintain CNS health, and whereas it has been implicated across different adult-onset neurodegenerative diseases, whether and how autophagy dysfunction might contribute to discrete facets of disease in the context of the adult and aging brain are uncertain. The overarching goal of this study was to determine how autophagy dysfunction evolves in the adult brain, and to explore whether any of the numerous functions ascribed to the adult brain might be preferentially vulnerable. We found that deactivation of autophagy in the adult animal resulted in the accumulation of a discrete subset of proteins in the synaptic compartment of neurons, and that the resulting accumulation of these proteins preferentially led to cognitive changes as opposed to locomotor ones. Moreover, upon comparison with a mouse model of AD, we observed biochemical changes reminiscent of those seen after global autophagy deactivation. Of note, the pattern of protein accumulation suggests that the reliance on autophagy is both cargo- and cell type-dependent.

A notable feature of this study is that we find both compartmental and temporal considerations must be made when exploring autophagy and the impact of its loss on the adult brain. For example, the biochemical analyses of whole tissue homogenates masked the progressive protein accumulation observed in the synaptosome compartment. Indeed, at the tissue level, protein accumulation also changed, demonstrating an initial accretion followed by reversal. This study revealed how alternative protein degradation pathways can respond to autophagy deactivation at the whole tissue level, whereas in the synaptosomal fraction, levels of affected proteins continued to rise over the course of the study, suggesting that the mechanisms compensating for the loss of autophagy in the cell as a whole are not able to compensate for the loss of autophagic function at the synapse.

A temporal consideration was also critical when examining the behavioral impact of the loss of autophagy, suggesting that autophagy deactivation need not yield immediate and catastrophic results. Our behavioral analysis did not demonstrate an immediate impact of autophagy on cognitive tasks, which is consistent with the recent report by Kallergi and colleagues, which found no change in initial learning of a task 2 weeks after neuron-specific autophagy deactivation using a similar iKO system [80]. Instead, we found it required greater time, such that cognitive dysfunction occurred after 6.5 wks. Our data suggest that the loss of autophagy at the synapse is initially tolerable, whereas only with time could the resulting change in protein homeostasis impair memory-dependent behaviors. These findings suggest that active adjustments in the regulation of synaptic protein degradation by autophagy might not be necessary for synaptic plasticity as previously suggested [13,80]. Instead, our data suggests that the role of autophagy at the synapse is primarily homeostatic. Given that cognitive dysfunction appeared in the absence of changes in hippocampal cell or synapse numbers, or neuroinflammation, these data rather support findings indicating that the maintenance of protein homeostasis at the synapse is essential not only for brain function, but may have a higher functional importance in the maintenance of brain circuits underlying learning and memory than overall cell health [2].

Given the preferential vulnerability of cognitive function over locomotor function to the loss of autophagy in the adult brain, we must reflect these findings in the context of adult-onset neurodegenerative diseases. Broadly across these varied disorders, autophagy dysfunction has been implicated as a root cause [51]. Our findings suggesting preferential vulnerability of cognition may be especially important for AD and possibly other disorders of cognition. Comparison to the autophagic defects in the APP PSEN1 double transgenic model [81] suggests that the diminished AV maturation present in these mice (a common feature across APP PSEN1 mouse models [33,37,82]) can lead to discrete changes in autophagy, such as the inability to properly capture synaptic cargoes such as SLC17A7/Vglut1, which could lead to its abnormal accumulation. Given that the dysfunction we find in this stage of the AD mice is milder than outright autophagy deactivation (as reflected in the lack of SQSTM1/p62 accumulation), it is possible that as time progresses, other proteins may also begin to accumulate, such as DLG4/PSD-95 and SYT1 which are reported to be transported through the autophagic pathway [8,9,11,12]. Since the dependence on autophagy will vary for the different proteins, future studies performing a more global analyses across multiple time points may be informative.

Much of the interest in autophagy and its role in the CNS has been informed by landmark studies eliminating autophagy throughout the development of the neuronal lineage [45,46,48,49]. Although incredibly informative, the flipside of these studies is a tendency to assume that all cells of the brain rely on autophagy similarly. It is clear from the ever-increasing number of studies in autophagy and the CNS, this is not at all the case [51]. The difference in behavioral outcomes upon autophagy deactivation, as well as the difference in protein turnover between SLC17A7/Vglut1 and SLC32A1/Vgat reflect these differences. It is therefore important to note, that although we focus on the hippocampus, these observations cannot be generalized to other brain regions. One brain region that has been more extensively studied is the cerebellum, where early changes have been reported by Komatsu, Yue, White and others [45,47,83]. It is likely that should we allow for longer deactivation in the *atg7*iKO mice, cell loss in the cerebellum would appear. Similarly, neuroinflammation might appear [84]. Finally, although we explored an impact of the loss of autophagy in hippocampal astrocytes and microglia in this study, given the very limited understanding of autophagy in different glial cells, how autophagy deactivation and dysfunction might or might not interfere with their function remains to be explored.

As a final note, in Figure 1, we found there to be delay in the loss of LC3-II in the synaptosome fraction, despite the loss of ATG7 expression. Given the clear accumulation of SQSTM1/p62 already present at that time, we felt the likely interpretation is that the persistence of autophagic membrane in the synaptosomes was due to either a lack of trafficking and degradation or recycling by ATG4. Nonetheless, the persistence of the autophagic membrane could lead to continued cargo-capture and help to preserve memory function in the *atg7*iKO (3.5 wks) mice. Future studies better identifying the membrane source upon which MAP1LC3/LC3 is lipidated, as well as using orthogonal approaches upon to deactivate autophagy, such as the loss of RB1CC1/FIP200 can help to further define why the persistence in LC3-II occurs.

Together, our data support prior evidence that autophagy is disrupted in AD, and indicate that this disruption leads to impaired synaptic protein homeostasis. If inadequate autophagy allows for a gradual dysregulation of synaptic homeostasis, this may explain the typically late-onset and slowly progressive nature of cognitive decline in AD. However, the isolated loss of autophagy in our *atg7*iKO model differs from the disruption that occurs in AD, likely with regards to both the mechanism of autophagy disruption, and the context in which it occurs. While our data emphasizes the specificity of autophagic processing of synaptic proteins, it also demonstrates the importance of the context of the disruption, as our *atg7*iKO mice were able to compensate to a degree to the autophagy deactivation, as with increased proteasome activity, which may not be as easily attainable in the brains of patients with AD, in whom multiple cellular functions are disrupted. If the disruption of autophagosome maturation in AD leads to an inability to properly capture and degrade autophagic cargoes, understanding how this contributes to the broader pathophysiology of AD may help direct the search for disease modifying treatments. Future studies will be needed to determine the mechanisms by which autophagy selectively degrades proteins at the synapse, how compensatory pathways are triggered when autophagy is inadequate, and where within the autophagic pathway we might target in order to specifically modify autophagy for neuroprotection in AD.

## Materials and methods

### Animals

All experiments were carried out in a manner consistent with National Institutes of Health guidelines and were approved by the Columbia University Institutional Animal Care and Use Committee (IACUC; AABM6572, AABG2560, AABL5571). Mice were housed under 12/12-h light/dark cycle with up to five mice per cage, maintained sex-segregated in a temperature-controlled environment and with access to food and water ad libitum. Experiments were all performed blind to genotype by using ear punching for identification. Genotyping was performed on tissue obtained from ear punching as follows: the tissue was first digested using proteinase K (Sigma-Aldrich, P6556; digestion buffer: 10 mM Tris HCl, pH 8.0, 100 mM NaCl, 10 mM EDTA, and 0.5% SDS in water). Samples were then ethanol precipitated to extract DNA. PCR was run on each sample using DreamTaq Green PCR Master Mix (2X) (ThermoFisher, K1081) in the Eppendorf AG 22,331 Hamburg Mastercycler.

### Atg7^fl/fl^:CAG^CrERTM^ mice

Atg7^fl/fl^ mice [53] were a kind gift from Dr. Masaaki Komatsu and were crossed with mice containing a tamoxifen (**tam**)-inducible Cre under the chicken *ACTB* promotor (CAG^CreERTM^) [54] (The Jackson Laboratories 004,682), to generate *atg7*iKO mice

### APP PSEN1 double-transgenic mice

APP PSEN1 mice were a generated in the Arancio lab (Columbia University Irving Medical Center, New York, NY) by crossing mice expressing transgenic human APP (K670N:M671L) [85] (with permission of K.H. Ashe) with mice expressing transgenic human PSEN1 (M146L, line 6.2) [86] to generate heterozygous double transgenic animals [73,87].

### Tamoxifen treatment

Tamoxifen (Sigma‐Aldrich, T5648) was prepared at 20 mg/mL in a solution containing 10% ethanol in corn oil, by heating at 37°C for 4 h and vortexing frequently. Between 4 and 5 months of age, littermate *Atg7*^*fl*/fl^ and *atg7*iKO mice were treated with this tamoxifen solution (75 mg/kg) via intraperitoneal injection for 5 days.

### Immunostaining

Immunostaining was performed as previously described [76]. Briefly, mice were deeply anesthetized with isoflurane and depth of anesthesia was confirmed with toe pinch and tail pinch. They were then transcardially perfused with 15–20 ml of 1× phosphate-buffered saline (PBS: 137 mM NaCl, 2.7 mM KCl, 10 mM Na_2_HPO_4_ [dibasic], and 1.8 mM KH_2_PO_4_ [monobasic]) followed by 15–20 ml of 4% paraformaldehyde in PBS. Brains were removed and post-fixed in 4% paraformaldehyde for 24 h, followed by incubation in 15% sucrose (Sigma-Aldrich, S5016) in 1× TBS (100 mM Tris-HCl, pH 7.4 and 150 mM NaCl) for 24 h, and finally 30% sucrose in 1X TBS for at least 24 h. In preparation for sectioning, brains were snap-frozen in 2-methylbutane in powdered dry ice then cryoprotected in OCT embedding medium (VWR 25,608–930). Tissue was sectioned at 30 μm using a Leica CM 1950 cryostat and stored at 4°C in 1× TBS 0.02% sodium azide as a preservative. Representative sections throughout the forebrain, spaced at 360 μm, were probed with antibodies against SQSTM1/p62 (Abcam, ab56416); SYP/synaptophysin (Abcam, ab32127; 1:100), DLG4/PSD-95 (Invitrogen, 7E3-1B8; 1:450), and MAP2 (Abcam, ab5392; 1:2000); AIF1/Iba-1 (FUJIFILM Wako chemicals, 019–19741; 1:1000); or GFAP (Millipore Sigma, G3893; 1:500). For diaminobenzidine (DAB) staining of AIF1/Iba1 and GFAP, sections were first washed in 1× PBS followed by blocking in 10% BSA (Sigma-Aldrich, A7906) in PBS containing 0.02% Triton X-100 (VWR 97,062–208) for 1 h. They were then incubated overnight in primary antibody, washed again, and then for Iba-1 and GFAP, incubated in HRP-conjugated secondary antibody (ThermoFisher 31,460 [anti-rabbit] or 31,430 [anti-mouse]) at room temperature for 2 h. Signal was detected using DAB (10 mg/25 ml; Sigma-Aldrich, D5905) in 1× PBS containing 0.0018% H_2_O_2_. Following the DAB reaction, sections were mounted on glass slides, air-dried, and coverslipped with Permount mounting medium (VWR 100,496–550). For immunofluorescence with SQSTM1/p62, SYP, DLG4/PSD-95, and MAP2, TBS replaced PBS for the blocking and washing steps, and sections were permeabilized in 1×TBS containing 0.02% Triton X-100 prior to blocking. After primary antibody probing and initial washing, sections were incubated with fluorescent-tagged secondary antibodies in TBS containing 0.02% Triton X-100 for 2 h protected from light, then again washed. Sections were then mounted using Fluoromount G with Dapi (Fisher Scientific, 00-4959-52) and coverslipped.

### Microscopy analysis

30 µm serially-cut coronal brain sections (every 360 μm) were used for all microscopic analyses. Tissue was matched across brains during sectioning, by designating “set 1” as the representative set containing the section with the initial crossing of the anterior commissure. For each immunoprobe, a number between 1 and 12 was selected at random, and this set number was used for staining that epitope across all brains. For light microscopy, CA1 was identified as the granular layer immediately superior to the dentate gyrus, as based on the Paxinos and Watson mouse brain atlas. A Leica DM i8 inverted brightfield microscope was used to obtain Iba-1 and GFAP images. A minimum of 2 images per section from each of 2 sections per animal were obtained. ImageJ was used to calculate the total area of signal above noise in each image for GFAP staining. QuPath was used to quantify soma size, cell spread, soma roundness, and cell number of AIF1/Iba1-positive cells [88]. For immunofluorescence microscopy, CA1 of the hippocampus was identified under 20× magnification by the dense DAPI signal of the striatum pyramidal layer as based on the Allen Brain Atlas that showed vertical MAP2 labeling of the axons that narrowed with distance from the cell body. Confocal laser scanning microscopy (LSM 700 Zen, Zeiss) was used at 40× magnification in water immersion to obtain 3 images of CA1 of the hippocampus per section from each of 2 sections per animal. ImageJ was used as previously described [89] to identify and count puncta representative of SQSTM1/p62, SYP, and DLG4/PSD-95, as well as regions of colocalization between SYP and DLG4/PSD-95. Puncta that were included in counting demonstrated signal above noise and were filtered for sizes >0.1 µm^3^ and <0.8 µm^3^ with one pixel of overlap or more. For all probes, the outcome measure was averaged for all images per section, then of all sections per animal, and finally of all animals per group.

### Tissue preparation for detergent soluble and insoluble fractions

To prepare tissue lysates, mice were deeply anesthetized with Isoflurane then euthanized with cervical dislocation. They were then decapitated, and their brains were rapidly removed, then dissected into the left forebrain, right forebrain, or hindbrain, with the junction between the cortex/thalamus with the midbrain used as the dividing point. Tissue was placed in a microtube with 500 µl of 1× PBS containing 2× Halt protease inhibitor cocktail (ThermoFisher 78,429) and homogenized using a handheld microtube homogenizer. An equal volume of detergent (2% Triton X-100 in PBS) was added to bring the final detergent concentration to 1% Triton X-100 in PBS (PBS-Triton X-100). This solution was incubated on ice for 30 min, then spun in an Eppendorf 5417 R centrifuge at 106.1×g for 5 min. The supernatant of this spin (S1) is the whole homogenate with large debris removed. To segregate the Triton X-100 soluble versus insoluble fraction, S1 was centrifuged at 20,800×g for 5 min. The resulting supernatant (S2) represents the detergent soluble fraction, whereas the pellet (P2) the detergent insoluble fraction. P2 was suspended in PBS-Tx in 8 M urea (Bio-Rad 1,610,730) and incubated on ice for 15 min. This was then centrifuged at 20,800×g for 5 min, and if a small viscous pellet (nucleic acid) was detected, the supernatant (S3) was carefully removed to not disturb the pellet. S3 is the solubilized insoluble fraction. The protein concentration of relevant suspensions was quantified using the Bio-Rad DC protein quantification assay kit (Bio-Rad 5,000,111) according to manufacturer’s instructions.

### Synaptosomal preparations

Two independent synaptosomal preparations were performed as previously described [90,91]. Briefly, in the first method, used to generate samples depicted in Figures 1, 3C, and S2B one hemi-forebrain, isolated as described above, was homogenized in TEVP buffer (10 mM Tris base, 5 mM NaF, 1 mM Na_3_VO_4_, 1 mM EDTA, 1 mM EGTA with pH adjusted to 7.4) containing 320 mM sucrose on ice. Samples were then centrifuged at 800×g for 10 min to remove nuclei and large debris. The supernatant was next centrifuged for 15 min at 9200×g. The pellet, which contained crude synaptosomes, was rinsed and then resuspended in TEVP buffer containing 35.6 mM sucrose and allowed to incubate on ice to lyse the synaptosomes. In the second method, used to generate Figure S2A and C, one hemi-forebrain, isolated as described above, was homogenized in synaptosome homogenization buffer (320 mM sucrose, 20 mM HEPES – KOH, with pH adjusted to 7.4) on ice. Samples were then centrifuged at 1000×g for 5 min. The supernatant was next centrifuged for 15 min at 17,000×g. The pellet was resuspended in synaptosome homogenization buffer and then layered on a gradient of 5 ml of 0.8 M sucrose, 20 mM HEPES – KOH pH 7.4 on the top of 5 ml 1.2 M sucrose, 20 mM HEPES – KOH pH 7.4. This gradient was centrifuged at 54,000×g for 90 min. The band at the interface was the synaptosome fraction. This was collected and resuspended in synaptosome homogenization buffer, then centrifuged at 20,000×g for 30 min. The pellet was collected and precipitated in acetone overnight, then dried and solubilized in synaptosome lysis buffer (7 M urea, 2 M thiourea (ThermoFisher, A12828–36), 4% [wt:vol] CHAPS (ThermoFisher, B21927), 20 mM Tris, pH 7.4, 5 mM MgCl_2_, 50 mM DTT). All samples were then quantified using the DC protein quantification assay kit (Bio-Rad 5,000,111).

### Autophagic vacuole isolation

Autophagic vacuoles were isolated based on protocols previously described [74–77,92]. In brief, brain tissue was rapidly dissected after mice were sacrificed and brains of mice from matched genotypes were pooled to obtain 10 g of brain tissue. This pooled brain tissue was then homogenized in ice-cold sucrose homogenization buffer (SHB; 0.25 M Sucrose, 10 mM HEPES pH 7.3, and 1 mM EDTA) containing protease inhibitors. The homogenate was centrifuged at 805×g for 10 min at 4°C to remove nuclei and large debris, and the supernatant was collected. This fraction was the post-nuclear supernatant (PNS). The PNS was then layered on top of a Histodenz (Sigma-Aldrich, D2158) gradient of 22.5% Nycodenz in SHB on the bottom and 9.5% Nycodenz above, and the gradient centrifuged at 144,428×g for 1 h at 4°C. All layers, including the AV/ER fraction, were collected. The AV/ER fraction was then layered onto a Nycodenz-Percoll gradient, which consisted of 22.5% Nycodenz on the bottom and 33% Percoll (Sigma-Aldrich, P1644) in SHB above, and centrifuged at 77,670×g for 30 min at 4°C. This separated out the AV layer from the ER layer. Percoll was then removed from that fraction using an Optiprep (Sigma-Aldrich, D1556) gradient: Optiprep was mixed with the AV sample in a 10:7 ratio, then 30% Optiprep in SHB was layered on top of the mixed sample, and a layer of SHB was added above that. Samples were centrifuged at 74,871×g for 30 min at 4°C, after which the purified AV fraction was collected. Protein concentration was measured using the Bradford protein quantification assay, (Bio-Rad 5,000,001).

### Western blotting (SDS-PAGE)

5–20 µg of protein was loaded onto a NuPAGE 4%-12% bis-tris gel (ThermoFisher, NP0335 [10-well] or NP0322 [12-well]) separated using gel electrophoresis, transferred to PVDF membranes (Invitrogen, LC2005), and blocked with 3% BSA at room temperature for 1 h. Membranes were incubated overnight with primary antibodies against SQSTM1/p62 (Abcam, ab56416; 1:2000), LC3-II (Abcam, ab48394; 1:1000), SYN1/synapsin I (Synaptic Systems 106 011; 1:2000), SYT1 (Sigma, MAB5200; 1:1500), SNAP25 (Cell Signaling Technology, 5308 [D7B4]; 1:1000), SLC17A7/Vglut1 (Synaptic Systems, 135 302; 1:1000), VAMP2 (Chemicon, Ab5856; 1:1000), SLC32A1/VIAAT (Thermo-Fisher, PA5–27569; 1:1000), SYP/synaptophysin (Abcam, ab14692; 1:2000), GRIN2B/NMDAR2b (Sigma, M265; 1:1000), DLG4/PSD-95 (Thermo-Fisher, MA1–046; 1:1000), ATG7 (Abcam, ab232348, 1:1000), or VCL/vinculin (Invitrogen 700,062; 1:1000). The membranes were washed 3 times and incubated with the appropriate horseradish peroxidase – conjugated secondary antibodies (ThermoFisher 31,460 [anti-rabbit] or 31,430 [anti-mouse]) at 1:500 to 1:3000. Membranes were developed with Clarity Western ECL substrate (Bio-Rad 1,705,061) and protein bands were detected using the BioRad VersaDoc imaging system. Band intensities were analyzed using ImageJ software and normalized to the appropriate loading control.

### Proteasome assay

The proteasome activity was measured on fresh frozen forebrain tissue using the Proteasome-Glo^TM^ Assay (Promega, G8531) to measure chymotrypsin-like activity, according to the manufacturer’s instructions.

### RT-qPCR

To perform RT-qPCR, RNA was first isolated from fresh frozen forebrain tissue using Trizol reagent (Thermo-Fisher, 15-596-026) per the manufacturer’s instructions. RNA concentration and purity was assessed using a nanodrop, and we proceeded with samples in which the A260:A280 ratio was > 1.8. cDNA libraries were then generated from these samples using SuperScript III Reverse Transcriptase (ThermoFisher 18,080–044) and random hexamers (Qiagen 79,236) according to the manufacturer’s instructions. The reverse transcription reaction parameters were the following: 25°C for 5 min → 50°C for 60 min → 70°C for 15 min. qPCR was then run on these cDNA libraries on QuantStudio 3 (Applied Biosystems by ThermoFisher), using iTaq™ Universal SYBR® Green Supermix (Bio-Rad 1,725,121), according to the manufacturer’s instructions. The qPCR parameters were: 95°C for 10 min → (95°C for 15 s → 60°C for 1 min)x40 → 95°C for 15 s → 60°C for 1 min → 95°C for 1 s. *Actb/β-actin* was used for normalization. The primer pairs used were as follows:

- Actb: forward – CAG TTC GCC ATG GAT GAC GAT; reverse – GCA GCT CAT TGT AGA AGG TGT

- Vamp2: forward – TCA AGC GCA AAT ACT GGT GG; reverse - GGG CTG AAA GAT ATG GCT GAG A

- GRIN2B/NMDAR2b: forward – TGC TAC AAC ACC CAC GAG AA; reverse - CTC CTC CAA GGT AAC GAT GC

- SLC17A7/Vglut1: forward – GAG CGC CAA GCT CAT GAA CC; reverse - GCC GTA GTG CAC CAG GGA G

- Snap25: forward – CTG GCA TCA GGA CTT TGG TT; reverse - GAT TTG GTC CAT CCC TTC CT

- SLC32A1/Vgat: forward – ACA AAC CCA AGA TCA CGG CG; reverse - CGA TGA GGA TCT TGC CGG TG

- Syn1: forward – AGG TTG AAG GCA TTG GTC AGA G; reverse - AGA AAC CCA GCC AGG ATG TGC

- DLG4/PSD-95: forward – TTT AAC CTT GAC CAC TCT CGT C; reverse - TCA AGC GCA AAT ACT GGT GG

- Syt1: forward – AGA CAT GGA CGT GGT GAA TCA; reverse - ACT CTC CGT CTT GTT GGC AC

- Syp: forward – GGC GTC GTT CTT GCC AAT CT; reverse - AGA TTC ACC TGA TGC AGA ACG G

QuantStudio Design&Analysis software v.1.5.1 calculated the C_T_ during the run, which was then used to calculate the delta C_T_, delta-delta C_T_, and the relative quantification (RQ), as previously described [93].

## Behavioral testing

### Radial arm water maze

The 2-day radial arm water maze was performed as previously described [66]. Briefly, mice were first trained to find a platform submerged in opaque water in a pool with 6 arms, then tested on their ability to find the platform based on its location within the room. The platform remained in the same arm of the pool for each trial for each mouse, and the mouse was placed in one of the other 5 arms in a random sequence, so that they were started in a different arm each trial than in any of the previous 4 trials. During the training phase, on alternate trials a marker was placed on the platform as a visual cue for the mouse as to its location. After the training phase, mice were tested on their ability to find the platform by removing the visual cue indicating the location of the platform for all subsequent trials. Mice were allowed 1 min to find the platform, during which time errors, as defined as an entry into an incorrect arm or not moving to a new part of the maze within 10 s, were counted. If the mouse did not find the platform by the end of one min, it was placed on the platform and allowed to remain for 15 s before being returned to its home cage. On day 1, mice were trained for 9 trials then tested for 6 trials. On day 2, they were tested for 15 trials. On both days, mice had a minimum of 1 h rest every 6 trials. Trials were averaged into 3 trials per block for data analysis.

### Barnes maze

The Barnes maze was performed as previously described [67,68]. Briefly, mice were first led to an escape hole on a 91 cm diameter, well-lit, circular, raised platform containing holes around the perimeter (Stoelting 60,170). Of those holes, 1 led to an escape box, and the others dead-ended. Next, over the course of 3 days, the mice had 5 training trials in which they were allowed to explore the platform for up to 120 s to find the escape hole. During this time, Stoelting ANYmaze software was used to track the latency to go to the escape hole. After a 48-h intertrial interval, mice were then tested on their ability to find the escape hole based on its location within the room, and latency to reach the escape hole was again measured.

### Open field

Mice were placed in the open field arena (43.2 cm × 43.2 cm × 30.5 cm) and their total distance traveled was measured for 10 min using equipment and software from Med Associates.

### Visible platform

Mice were placed in an open pool containing a platform. Ethovision software was used to monitor their swim speed over 1 min or until they found the platform for 6 trials/day on 2 consecutive days. On both days, animals had a minimum of 1 h rest after the first 3 trials and average speed was calculated for each day.

### Statistics

All results in APP PSEN1 mice were analyzed using a 2-tailed student’s t-test. With the exception of the 2dRAWM, the Barnes maze, and the visible platform test, all results in autophagy deactivation studies were analyzed using a one-way ANOVA with duration of autophagy deactivation as the independent variable. The 2dRAWM, Barnes maze, and the visible platform test were analyzed using a mixed model ANOVA to account for the repeated measures. Tukey’s post-hoc comparison was used to further interrogate significant comparisons when there were equal variances, and Games-Howell test was used for unequal variances. Please see Tables S1 and S3 for full details of statistical results.

## Supplementary Material

Supplemental Material
