# Supplementary material for: Adult-onset deactivation of autophagy leads to loss of synapse homeostasis and cognitive impairment, with implications for alzheimer disease
Source: Autophagy. 2024 Jul 1;20(11):2540–55. doi: 10.1080/15548627.2024.2368335 (PMC11572145; doi:10.1080/15548627.2024.2368335)
Supplement: Supplemental Material [file KAUP_A_2368335_SM7234.docx]

**Adult-Onset Deactivation of Autophagy Leads to Loss of Synapse Homeostasis and Cognitive Impairment, with Implications for Alzheimer disease**


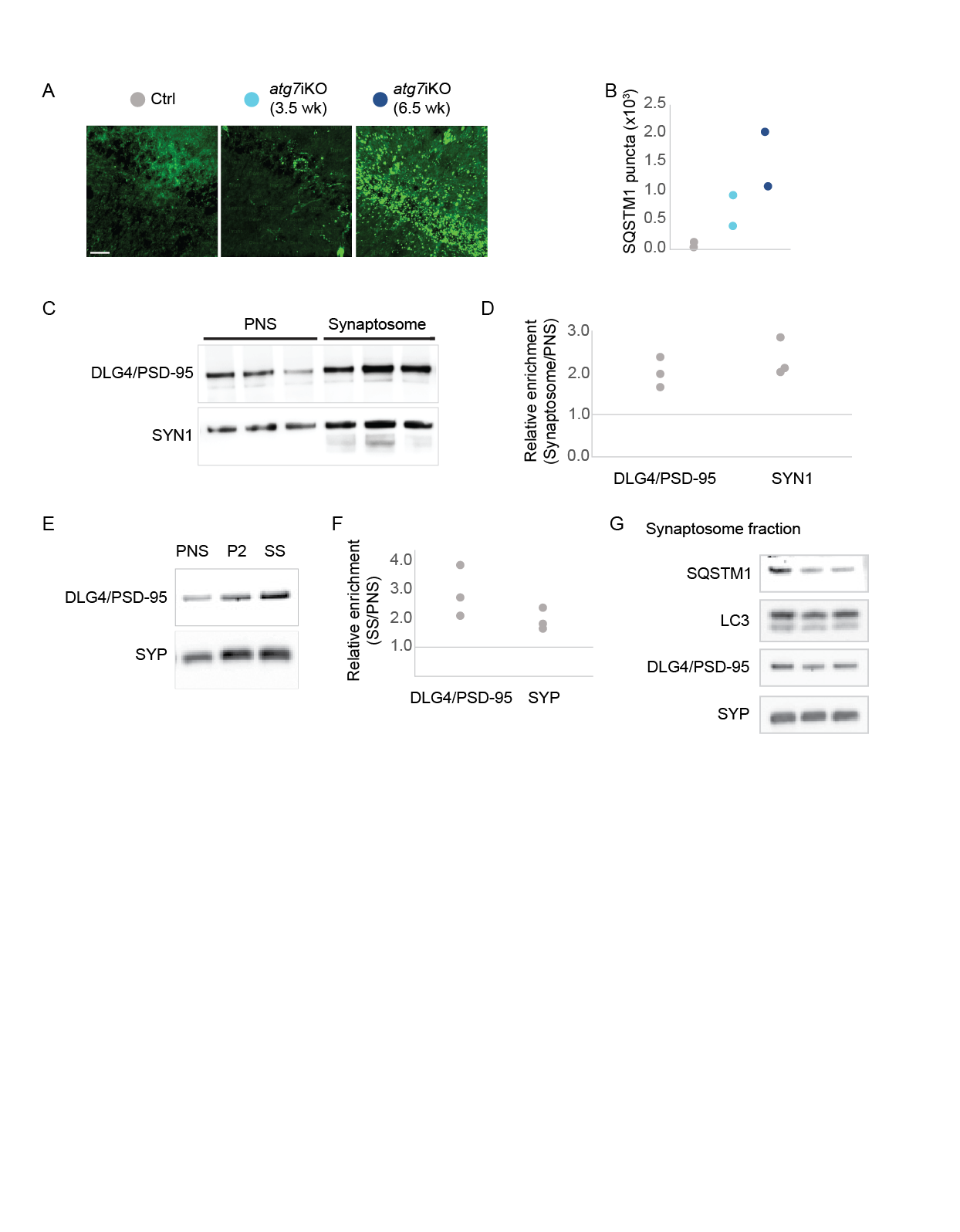


**Figure S1**. Autophagy deactivation and synaptic autophagy. (**A** and **B**) Confirmation of autophagy deactivation by monitoring SQSTM1/p62 accumulation. (**A**) Hippocampal brain sections were probed for SQSTM1/p62, demonstrating an accumulation of puncta following autophagy deactivation. Scale bar represents 20 μm. (**B**) Quantification of the number of puncta from CA1 of the hippocampus. (**C-G**) Using synaptosome fractionation to study autophagy at the synapse. (**C** and **D**) 10 μg of the post-nuclear supernatant (PNS) and synaptosomal fraction represented in Figures 1 and 3 were subjected to quantitative western blot analysis and probed for the canonical pre- and post-synaptic proteins SYN1 and DLG4/PSD-95, respectively. Relative enrichment is calculated as the relative density of the protein of interest in the synaptosome fraction/PNS. Values greater than 1 indicate enrichment. N=3 independent preparations. (**E** and **F**) An independent “float-up” synaptosome preparation also suggests the presence of autophagosomes at the synapse. Forebrain homogenates of wildtype mice were subjected to a fractionation method that enriches for pre- and post-synaptic membrane (Gulyassy et al, method #2). 10 μg of PNS, the crude synaptosome fraction (P2) and purified synaptosome fraction (SS) were subjected to western blot analysis and probed for SYP and DLG4/PSD-95. Relative enrichment in SS was calculated as described in D. (**G**) Similarly to the method used for Figures 1 and 3, SQSTM1/p62 and MAP1LC3/LC3 are found in pre- and post-synaptic enriched synaptosome fractions. n=3 independent preparations.

**
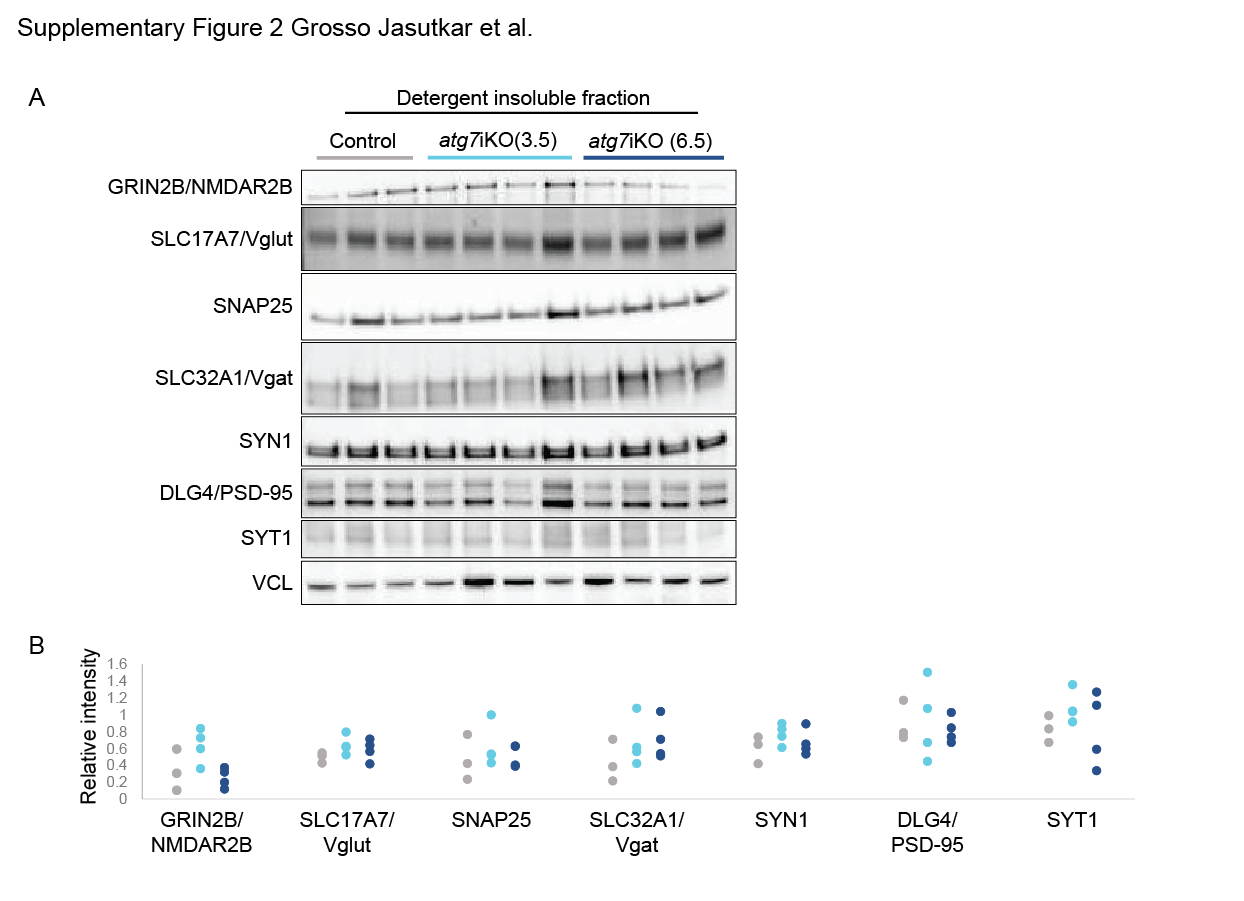
**

**Figure S2.** Synaptic proteins do not accumulate in the insoluble fraction of *atg7*iKO forbrain homogenates following autophagy deactivation. (**A**) The forebrains of *atg7*iKO mice were homogenized and the insoluble fraction was subjected to quantitative western blot that was then probed for multiple synaptic proteins. Analysis of these results demonstrated no accumulation of any of the synaptic proteins evaluated for in the insoluble fraction. VCL serves as a loading control. (**B**) Quantification of A. Individual values of relative intensity shown (n=3 control, 4 *atg7*iKO (3.5 wks), and 4 *atg7*iKO (6.5 wks).

**
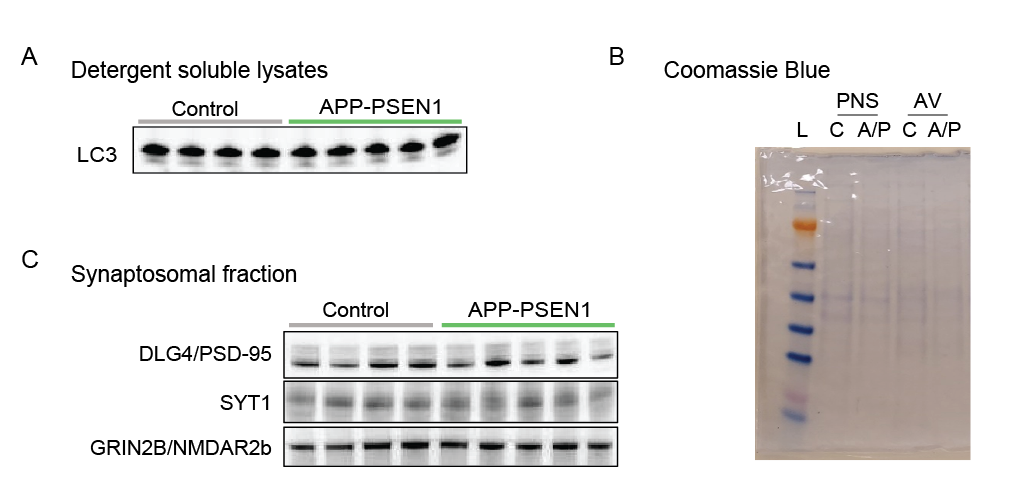
**

**Figure S3.** Additional analyses of APP-PSEN1 double transgenic mice. (**A**) Immunoblot analyses of detergent soluble lysates from mouse brain for MAP1LC3/LC3, as part of Figure 7A and 7G. No notable difference across genotype was observed in LC3-II levels. n=4 control, n=5 APP-PSEN1. (**B**) Representative image of Coomassie Blue staining of PNS and AV fractions to confirm loading in Figure 7C. These gels are run to ensure loading. n=3. A/P = APP-PSEN1; C = Ctrl; L = Ladder (**C**) The concentration of synaptic proteins does not change in the synaptosomal fraction of APP PSEN1 double transgenic mice. The APP PSEN1 mouse forebrain was enriched for the synaptosomal fraction and subjected to quantitative western blot. This demonstrated no accumulation of synaptic proteins. n=4 control, n=5 APP PSEN1.

# SUPPLEMENTARY Tables

| Table S1. Description of statistical test applied can be found in Materials and methods. | | | | | | |
| --- | --- | --- | --- | --- | --- | --- |
| Figure panel | **Subject** | **F- value** | **p-statistic** | **df within groups** | **df between groups** | **Post-hoc comparisons (mean**±St.Dev)  **(n.s. = no significance detected)** |
| FIGURE 1 | | | | | | |
| 1C | ATG7 | 49.749 | <0.001 | 6 | 2 | Ctrl (1.8524 ± 0.24256) vs *atg7*iKO(3.5) (0.2152 ± 0.01869), p<0.001 |
|  |  |  |  |  |  | Ctrl vs *atg7*iKO(6.5) (0.0574 ± 0.01974), p<0.001 |
| 1E | SQSTM1/p62 | 67.484 | <0.001 | 8 | 2 | Ctrl (0.3648 ± 0.04435) vs *atg7*iKO(3.5) (0.9142 ± 0.04729), p <0.001 |
|  |  |  |  |  |  | Ctrl vs *atg7*iKO(6.5) (1.1753 ± 0.04935), p <0.001 |
|  |  |  |  |  |  | *atg7*iKO(3.5) vs *atg7*iKO(6.5), p = 0.010 |
| 1G | LC3-II:LC3-I ratio | 5.831 | 0.027 | 8 | 2 | Ctrl (0.795 ± 0.058) vs *atg7*iKO(6.5) (0.4922 ± 0.078), p=0.024 |
|  | SQSTM1/p62 | 65.556 | <0.001 | 8 | 2 | Ctrll (0.795 ± 0.058) vs *atg7*iKO(3.5)(0.690 ± 0.040) p = 0.002 |
|  |  |  |  |  |  | Ctrl vs *atg7*iKO(6.5)(0.4922 ± 0.078) p < 0.001 |
|  |  |  |  |  |  | *atg7*iKO(3.5) vs *atg7*iKO(6.5)p < 0.001 |
| FIGURE 2 | | | | | | |
| 2B | VAMP2 | 2.65 | 0.131 | 8 | 2 | n.s. |
|  | GRIN2B /NMDAR2b | 5.553 | 0.031 | 8 | 2 | Ctrl (0.833 ± 0.116) vs *atg7*iKO(3.5)(1.217 ± 0.031), p = 0.033 |
|  | SLC17A7 /Vglut1 | 8.979 | 0.009 | 8 | 2 | Ctrl (1.031 ± 0.038) vs *atg7*iKO(3.5)(1.306 ± 0.068), p = 0.019 |
|  |  |  |  |  |  | *atg7*iKO(3.5)vs *atg7*iKO(6.5)(1.037 ± 0.040), p = 0.014 |
|  | SNAP25 | 4.64 | 0.046 | 8 | 2 | n.s, |
|  | SLC32A1/Vgat | 0.721 | 0.516 | 8 | 2 | n.s. |
|  | SYN1 | 1.045 | 0.395 | 8 | 2 | n.s. |
|  | DLG4/PSD-95 | 8.065 | 0.012 | 8 | 2 | Ctrl (0.726 ± 0.168) vs *atg7*iKO(3.5)(1.228 ± 0.220), p = 0.011 |
|  | SYT1 | 6.398 | 0.022 | 8 | 2 | Ctrl (0.589 ± 0.091) vs *atg7*iKO(3.5)(1.017 ± 0.091), p = 0.020 |
| 2C | Chymotrypsin-like activity  (luminescence) | 8.769 | 0.01 | 8 | 2 | Ctrl (1899.5 ± 323.7977) vs *atg7*iKO(6.5)(3266.25 ± 324.4168), p = 0.026 |
|  |  |  |  |  |  | *atg7*iKO(3.5)(1570 ± 184.4478) vs 6.5 wk iKO, p = 0.013 |
| ­FIGURE 3 | | | | | | |
| 3B | VAMP2 | 9.697 | 0.007 | 8 | 2 | Ctrl (0.540 ± 0.033) vs *atg7*iKO(6.5)(0.736 ± 0.028), p = 0.006 |
|  | GRIN2B /NMDAR2b | 6.309 | 0.023 | 8 | 2 | Ctrl (0.667 ± 0.107) vs *atg7*iKO(3.5)(0.994 ± 0.053), p = 0.043 |
|  |  |  |  |  |  | Ctrl vs *atg7*iKO(6.5)(1.033 ± 0.075), p = 0.026 |
|  | SLC17A7 /Vglut | 7.716 | 0.014 | 8 | 2 | Ctrl (0.973 ± 0.029) vs *atg7*iKO(6.5)(1.012 ± 0.028), p = 0.023 |
|  | SNAP25 | 6.502 | 0.021 | 8 | 2 | Ctrl (0.668 ± 0.062) vs *atg7*iKO(6.5)(0.981 ± 0.035), p = 0.017 |
|  | SLC32A1/Vgat | 1.207 | 0.348 | 8 | 2 | n.s. |
|  | SYN1 | 1.044 | 0.395 | 8 | 2 | n.s. |
|  | DLG4/PSD-95 | 0.240 | 0.792 | 8 | 2 | n.s. |
|  | SYT1 | 7.230 | 0.016 | 8 | 2 | Ctrl (1.269 ± 0.094) vs *atg7*iKO(3.5)(0.898 ± 0.053), p = 0.013 |
| FIGURE 4 | | | | | | |
| 4B | Synapse count | 2.537 | 0.117 | 13 | 2 | n.s. |
| 4D | Cell number | 0.857 | 0.451 | 11 | 2 | n.s. |
| FIGURE 5 | | | | | | |
| 5B | Soma size | 0.551 | 0.606 | 5.236 | 2 | n.s. |
| 5C | Soma roundness | 3.562 | 0.068 | 10 | 2 | n.s. |
| 5D | Cell Spread | 3.062 | 0.088 | 10 | 2 | n.s. |
| 5E | No. of cells per area | 8.229 | 0.007 | 11 | 2 | Ctrl (2.732 x 10^-4^ ± 4.5 x 10^-5^) vs *atg7*iKO(6.5)(4.675 x 10^-4^ ± 4.2 x 10^-5^), p = 0.016 |
|  |  |  |  |  |  | *atg7*iKO(3.5)(2.321 x 10^-4^ ± 2.0 x 10^-5^) vs *atg7*iKO(6.5), p = 0.009 |
| 5G | GFAP Signal | 0.216 | 0.809 | 11 | 2 | n.s. |
| FIGURE 6 | | | | | | |
| 6A | Training | 0.811 | 0.451 | 44 | 2 | n.s. |
|  | Testing | 8.378 | <0.001 | 44 | 2 | Ctrl vs *atg7*iKO(6.5), p < 0.001 |
|  |  |  |  |  |  | atg7iKO(3.5) vs *atg7*iKO(6.5), p = 0.033 |
| 6B | Training | 8.076 | 0.03 | 18 | 2 | Overall: Ctrl vs *atg7*iKO(6.5), p = 0.002 |
|  |  |  |  |  |  | Overall: *atg7*iKO(3.5) vs *atg7*iKO(6.5), p = 0.030 |
|  | Test | 18.213 | <0.001 | 21 | 2 | Ctrl (19.263 ± 2.872) vs *atg7*iKO(6.5) (118.65 ± 39/211), p < 0.001 |
|  |  |  |  |  |  | *atg7*iKO(3.5) (5.2 ± .747) vs *atg7*iKO(6.5), p < 0.001 |
| 6C | Total Distance traveled | 0.006 | 0.994 | 50 | 2 | n.s. |
| 6D: | Swim speed | 5.174 | 0.010 | 38 | 2 | Ctrl vs *atg7*iKO(3.5), p = 0.021 |
| 6D: Day 1 |  | 6.782 | 0.003 | 38 | 2 | Ctrl (16.170 ± 0.700) vs *atg7*iKO(3.5) (12.950 ± 0.656), p=0.003 |
| 6D: Day 2 |  | 2.211 | 0.123 | 40 | 2 | n.s. |

| Table S2. RT-qPCR of synaptic proteins in *atg7*iKO mice. | | | | | |
| --- | --- | --- | --- | --- | --- |
| Gene | **Group** | **Mean RT** | **Standard Error** | **F-Statistic (2,7)** | **p-value** |
| V*amp2* | control | 0.8373 | 0.14648 | 0.898 | 0.45 |
|  | *atg7*iKO (3.5) | 1.0193 | 0.05254 |  |  |
|  | atg7iKO (6.5) | 0.9298 | 0.06356 |  |  |
| *Grin2b* | control | 1.0353 | 0.01812 | 4.823 | 0.048* |
|  | *atg7*iKO (3.5) | 0.754 | 0.0466 |  |  |
|  | *atg7*iKO (6.5) | 0.8415 | 0.07963 |  |  |
| *Slc17a7* | control | 0.945 | 0.02829 | 3.415 | 0.102 |
|  | *atg7*iKO (3.5) | 1.136 | 0.1286 |  |  |
|  | *atg7*iKO (6.5) | 0.8863 | 0.05523 |  |  |
| *Snap25* | control | 1.2427 | 0.1214 | 0.56 | 0.595 |
|  | *atg7*iKO (3.5) | 1.461 | 0.21907 |  |  |
|  | *atg7*iKO (6.5) | 1.297 | 0.09826 |  |  |
| *Slc32a1* | control | 0.9153 | 0.05657 | 0.089 | 0.916 |
|  | *atg7*iKO (3.5) | 0.887 | 0.16004 |  |  |
|  | *atg7*iKO (6.5) | 0.9513 | 0.09586 |  |  |
| *Syn1* | control | 1.1923 | 0.13117 | 3.737 | 0.088 |
|  | *atg7*iKO (3.5) | 0.904 | 0.036 |  |  |
|  | *atg7*iKO (6.5) | 0.888 | 0.05627 |  |  |
| *Dlg4* | control | 0.868 | 0.10797 | 0.339 | 0.724 |
|  | *atg7*iKO (3.5) | 0.7823 | 0.03117 |  |  |
|  | *atg7*iKO (6.5) | 0.788 | 0.07759 |  |  |
| *Syt1* | control | 1.088 | 0.07632 | 1.592 | 0.269 |
|  | *atg7*iKO (3.5) | 1.1663 | 0.15533 |  |  |
|  | *atg7*iKO (6.5) | 0.939 | 0.04774 |  |  |
| *Syp* | control | 1.0397 | 0.15056 | 0.422 | 0.672 |
|  | *atg7*iKO (3.5) | 0.8907 | 0.02728 |  |  |
|  | *atg7*iKO (6.5) | 0.9815 | 0.10848 |  |  |
| *A Tukey post-hoc test revealed that the difference between the groups was driven by the comparison between control (1.0353 ± 0.01812) and 3.5wk iKO (0.754 ± 0.0466), p = 0.045. | | | | | |

| Table S3. Statistical analysis (Student’s t-test) for Figure 7. | | | | |
| --- | --- | --- | --- | --- |
| Figure panel | **Subject** | **Control (Mean±SEM)** | **APP-PSEN1 (Mean±SEM)** | **p statistic** |
| 7A | LC3-II/LC3-I | 0.3403 ± 0.049 | 0.6959 ± 0.062 | 0.003 |
|  | insoluble SQSTM1/p62 | 0.5867 ± 0.075 | 0.866 ± 0.0115 | 0.098 |
|  | soluble SQSTM1/p62 | 0.793 ± 0.055 | 0.747 ± 0.084 | 0.676 |
|  | soluble SLC17A7/Vglut | 0.799 ± 0.013 | 0.876 ± 0.064 | 0.301 |
|  | soluble DLG4/PSD-95 | 0.910 ± 0.088 | 1.036 ± 0.110 | 0.418 |
|  | soluble SYT1 | 0.864 ± 0.049 | 0.794 ± 0.055 | 0.393 |
|  | insoluble SLC17A7/Vglut | 0.755 ± 0.087 | 1.159 ± 0.124 | 0.040 |
|  | Insoluble DLG4/PSD-95 | 0.285 ± 0.063 | 0.494 ± 0.114 | 0.159 |
|  | insoluble SYT1 | 0.727 ± 0.049 | 0.838 ± 0.089 | 0.350 |
